# Clinical application of somatosensory amplification in psychosomatic medicine

**DOI:** 10.1186/1751-0759-1-17

**Published:** 2007-10-09

**Authors:** Mutsuhiro Nakao, Arthur J Barsky

**Affiliations:** 1 Department of Hygiene and Public Health, Teikyo University School of Medicine, Tokyo, Japan; 2 Division of Psychosomatic Medicine, Teikyo University Hospital, Tokyo, Japan; 3 Department of Psychiatry, Brigham and Women's Hospital, Harvard Medical School, Boston, MA, USA

## Abstract

Many patients with somatoform disorders are frequently encountered in psychosomatic clinics as well as in primary care clinics. To assess such patients objectively, the concept of somatosensory amplification may be useful. Somatosensory amplification refers to the tendency to experience a somatic sensation as intense, noxious, and disturbing. It may have a role in a variety of medical conditions characterized by somatic symptoms that are disproportionate to demonstrable organ pathology. It may also explain some of the variability in somatic symptomatology found among different patients with the same serious medical disorder. It has been assessed with a self-report questionnaire, the Somatosensory Amplification Scale. This instrument was developed in a clinical setting in the U.S., and the reliability and validity of the Japanese and Turkish versions have been confirmed as well.

Many studies have attempted to clarify the specific role of somatosensory amplification as a pathogenic mechanism in somatization. It has been reported that somatosensory amplification does not correlate with heightened sensitivity to bodily sensations and that emotional reactivity exerts its influence on somatization via a negatively biased reporting style. According to our recent electroencephalographic study, somatosensory amplification appears to reflect some aspects of long-latency cognitive processing rather than short-latency interoceptive sensitivity.

The concept of somatosensory amplification can be useful as an indicator of somatization in the therapy of a broad range of disorders, from impaired self-awareness to various psychiatric disorders. It also provides useful information for choosing appropriate pharmacological or psychological therapy. While somatosensory amplification has a role in the presentation of somatic symptoms, it is closely associated with other factors, namely, anxiety, depression, and alexithymia that may also influence the same. The specific role of somatosensory amplification with regard to both neurological and psychological function should be clarified in future studies. In this paper, we will explain the concept of amplification and describe its role in psychosomatic illness.

## Assessment of stress-related conditions

Stress is the term used to define the body's physiological and/or psychological reaction to circumstances that require behavioral adjustment. According to the Japanese National Survey of Health in 2004 [1], 49% of those 12 years or older reported experiencing stress in their daily lives. In this survey, the subjects answered "yes" if they perceived stress in any of 28 domains including work, family and neighborhood relations as well as living-, social-, financial-, and health-related situations. A higher percentage of perceived stress was observed in women (53%) than in men (45%); the percentage of perceived stress has continued to increase over the years. in both sexes. Work-related problems were the most frequent stressors, followed by health-related and then financial problems [1]. One of the interesting findings of this national survey[1] was that stress was more frequently reported by those complaining of any physical or psychological symptoms; 69% of 37 million people with such symptoms reported stress as opposed to only 39% of 75 million people without symptoms who did (*p *< 0.0001, chi-square test). These results [1] suggest that those perceiving psychosocial stress are also likely to complain of mind/body symptoms.

The symptoms related to psychosocial stress are often temporary and disappear with the relief of such stress. However, a specific illness may be caused when the experienced stressors are too intense and persistent. When people are vulnerable to stress because of their character and ability to adapt, a psychosomatic illness is likely to occur even if the stressors are mild or moderate[2]. The Japanese Society of Psychosomatic Medicine defines psychosomatic illness as any physical condition with organic or functional damage affected by psychosocial factors in its onset or development[3]. This definition largely corresponds to that of "psychosocial factors affecting general medical conditions (code 316.00)" of the Diagnostic and Statistical Manual of Mental Disorders fourth edition, text revision (DSM-IV-TR) [4], published by the American Psychiatric Association.

## Somatization and psychosomatic illness

According to a study[5] of outpatients visiting a Japanese psychosomatic clinic (n = 1,432), the most common physical disorders observed were autonomic nervous dysfunction, irritable bowel syndrome, essential hypertension, and hyperventilation. Eating disorders, anxiety disorders, and depressive episodes were also prevalent. When the DSM-III-R or DSM-IV criteria were applied to the total sample, "somatoform disorders not otherwise specified" became the most common diagnosis, followed by bulimia nervosa, depressive disorders not otherwise specified, anorexia nervosa, conversion disorder, major depression or depressive disorder, panic disorder with agoraphobia, and psychological factors affecting physical (or medical) condition.

These findings appear to conflict with those from Western countries[6,7]. For example, a study in an Italian psychosomatic clinic [6] showed that the most frequent diagnosis was "psychological factors affecting physical condition," followed by affective illness, anxiety disturbance, and somatoform disorders according to the DSM-III criteria. In a Japanese study[5], a detailed manual of diagnoses was made, and the physicians specializing in psychosomatic medicine discussed the patients' diagnoses in order to improve the reliability of diagnoses; however, many patients were still categorized into "somatoform disorders not otherwise specified." These studies indicate that there is considerable confusion and ambiguity in diagnosing patients with somatization. To assess such patients more objectively, the concept of somatosensory amplification may be useful in clinical practices.

## Concept of somatosensory amplification

Somatosensory amplification refers to the tendency to experience a somatic sensation as intense, noxious, and disturbing [8]. The construct of somatosensory amplification is helpful in the assessment of somatization and in the conceptualization of psychosomatic illness [8-10]. Somatosensory amplification may have a role in a variety of medical conditions characterized by somatic symptoms that are disproportionate to demonstrable organ pathology. It may also explain some of the variability in somatic symptomatology found among different patients with the same serious nonpsychiatric medical disorder.

Studies of amplification in patients with somatoform disorders have been conducted. These studies have resulted in the standardization of the Somatosensory Amplification Scale (SSAS) checklist in 1990 [11]. (Table 1) The original SSAS[11] was developed in a clinical setting in the U.S., and the reliability and validity of the Japanese[12] and Turkish forms[13] of the SSAS have been confirmed as well. It is a 10-item self-report questionnaire, and the respondents rate the degree to which each statement is ''characteristic of you in general,'' on an ordinal scale of 1 to 5. A higher total score indicates greater symptom amplification (score range of 10 to 50).

**Table 1:** Somatosensory Amplification Scale

1.	When someone else coughs, it makes me cough too.
2.	I can't stand smoke, smog, or pollutants in the air.
3.	I am often aware of various things happening within my body.
4.	When I bruise myself, it stays noticeable for a long time.
5.	Sudden loud noises really bother me.
6.	I can sometimes hear my pulse or my heartbeat throbbing in my ear.
7.	I hate to be too hot or too cold.
8.	I am quick to sense the hunger contractions in my stomach.
9.	Even something minor, like an insect bite or a splinter, really bothers me.
10.	I have a low tolerance for pain.

According to our clinical experiences and previous studies targeting the Japanese population, SSAS scores over 30 may reflect a highly somatizing condition; the average SSAS scores were 24–29 in groups of university students[14], office workers[15], and outpatients visiting a general internal medicine clinic [10] whereas it was 32 in the patients visiting a psychosomatic clinic[10]. Based on such experimental and epidemiological studies, we believe that somatosensory amplification appears to have both trait-like and state-like properties[10,14,15].

The SSAS is useful in briefly and objectively evaluating patients with mind/body distress. The total number of reported somatic symptoms has been considered to be a powerful predictor of functional impairment in physical, psychological, and social functioning [16], and the SSAS scores were shown to be closely associated with the total number of somatic symptoms in patients visiting a psychosomatic clinic[10].

## Somatosensory amplification and alexithymia

Alexithymia is a personality construct derived from the clinical observation of patients with psychosomatic illness [17]. It is characterized by difficulty in distinguishing between emotions and bodily sensations, difficulty identifying and describing emotions, and a mechanistic, concrete, literal cognitive style. Evidence has suggested that alexithymia is associated with a tendency to develop functional somatic symptoms [18-20]. Our recent study reported that the SSAS was significantly correlated with a Toronto Alexithymia Scale (TAS), in the sample of individuals with psychosomatic illness[10]. High rates of alexithymia have been reported in patients with essential hypertension, myocardial infarction, inflammatory bowel diseases, functional gastrointestinal disorders, and chronic pain [21], and the close relationship between alexithymia and somatosensory amplification has been demonstrated in chronic pain [22] and functional dyspepsia[23]. The statistical and clinical association between somatosensory amplification and alexithymic characteristics appears logical. The roles of these two psychological concepts in clinical conditions should be further studied to clarify symptom generation and perception in patients with psychosomatic illness.

## Role of somatosensory amplification

Three components of somatosensory amplification have been described[24]: bodily hypervigilance that involves heightened self-scrutiny and increased attention to unpleasant bodily sensations; the tendency to select and focus on certain relatively weak or infrequent sensations; and the tendency to appraise ambiguous or vague visceral and somatic sensations as abnormal, pathological, and symptomatic of disease, rather than considering them to be normal. This cognitive appraisal causes alarm and anxiety in relation to the perception of symptoms and is proposed to act as the intermediary between the perception of bodily sensations on one hand and hypochondriacal beliefs and behaviors on the other. Many studies have attempted to clarify the specific role of somatosensory amplification as a pathogenic mechanism in somatization [25-27]. A recent study[28] failed to find a significant relationship between the SSAS and heartbeat detection ability and interoceptive sensitivity, suggesting that self-reported somatosensory amplification does not correlate with objectively measured sensitivity to bodily sensations. In another study[29], emotional reactivity appeared to exert its influence on somatization via a negatively biased reporting style and not via somatic sensitivity.

**Figure 1:**
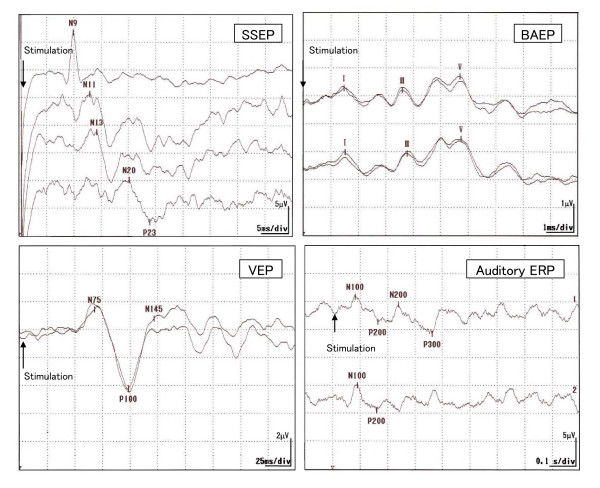
Short-latency somatosensory evoked potential (SSEP), brainstem auditory-evoked potential (BAEP), visual evoked potential (VEP), and auditory event-related potential (ERP) recorded in human.

To elucidate the link between somatosensory amplification and sensitivity to bodily sensations, we conducted an electroencephalographic (EEG)[14] study in 33 university students examining the relationship between somatosensory amplification and four different types of evoked potentials, i.e., short-latency somatosensory evoked potential (SSEP), brainstem auditory-evoked potential (BAEP), visual evoked potential (VEP), and auditory event-related potential (ERP). (Figure 1) We found that the SSAS was significantly associated with the parameters of auditory ERP (i.e., the P200 latency and P300 amplitude) after adjusting for the effects of the TAS and of the depression and tension-anxiety subscales of the Profile of Mood States[14].(Table 2) This significant relationship between the SSAS and auditory ERP appears important. The SSEP (normally 8.0–30.0 ms in latency) reflects mechanical processing in short pathways from sensory-organ to the primary cortex, whereas auditory ERP (normally 100–350 ms in latency) reflects cognitive processing of bodily sensations which they operationally define as processing in long pathways from the sensory-organ to cerebral cortex via complex synaptic circuits[30,31]. Based on the insignificant findings of SSEP in the study[14], the SSAS was not suggested to be a measure of mechanical conduction from the sensory organs to the first sensory cortex areas. Rather the SSAS seems to be more closely related to the processing of sensory input at higher level of central nervous system. Auditory ERP is divided into early- (<100 ms) and late- (>100 ms) occurring components [30,31]. The late component represents aspects of information processing, such as attention allocation and activation of immediate memory, while the early component represents the activity of the sensory nerves, brainstem, and primary sensory cortex. Thus a delayed P200 and diminished P300 amplitude may reflect a disturbance in the awareness of or the attention paid to afferent stimuli due to abnormally increased levels of physiological inhibition, possibly at the level of the brainstem, cortex, or both [32,33]. Although the findings [14] should be viewed as preliminary, somatosensory amplification appears to reflect some aspects of long-latency cognitive processing rather than short-latency interoceptive sensitivity from the viewpoint of EEG.

**Table 2:** Evoked potentials associated with the SSAS

EEG variables	Means (S.D.) and coefficient^a ^to SSAS (signed)			
	
	Latency, msec		Amplitude, μV	
Somatosensory evoked potential				
N9	9.5 (0.7)	(+)	5.1 (2.3)	(-)
N9–N13	3.8 (0.6)	(+)	1.8 (0.8)	(-)
N13–N20	5.8 (1.2)	(-)	1.1 (0.7)	(+)
N20–P23	3.3 (1.2)	(-)	1.2 (0.7)	(+)
Auditory evoked potential				
I	1.5 (0.1)	(+)	0.2 (0.1)	(-)
III	3.7 (0.1)	(-)	0.3 (0.1)	(-)
V	5.6 (0.2)	(+)	0.6 (1.3)	(-)*
Visual evoked potential				
N75	73.2 (10.1)	(-)	3.3 (2.3)	(-)
P100	103.1 (10.3)	(+)*	5.9 (2.3)	(-)
N145	138.0 (15.9)	(-)	3.3 (1.7)	(-)
Event-related potential				
N100	111.5 (40.6)	(+)	4.2 (2.1)	(-)
P200	180.3 (45.2)	(+)**	2.9 (1.6)	(+)
N200	248.1 (51.7)	(+)	4.4 (2.9)	(-)
P300	333.6 (70.7)	(+)	2.7 (1.8)	(-)**

^a ^The coefficient refers to the partial Pearson's correlation coefficient adjusted for the Toronto alexithymia scale scores and depression and tension-anxiety scores on the Profile of Mood States. A positive (negative) mark indicates a positive (negative) coefficient; *p < 0.10 and **p < 0.05.

## Use of SSAS

The concept of somatosensory amplification enables us to quantify the intensity of various somatic symptoms that patients complain about, eliminating the subjective judgment of physicians. The objective measurement of somatosensory sensitivity with a psychophysiological instrument is difficult and time consuming. The SSAS is simple and requires less than 10 min to complete. Although there are many reliable questionnaires for assessing somatoform symptoms, such as the Whitely Index, Somatic Symptom Inventory, the hypochondriasis subscale on the Minnesota Multiphasic Personality Inventory, and the somatization scale on the Symptom Checklist 90R [34-37], the SSAS enables the evaluation of somatosensory amplification in various diseases with fewer questions.

The SSAS should be used in combination with other psychological questionnaires in a test battery. This is because mood states, psychosocial stress, and the number of somatic symptoms can all influence somatosensory amplification as shown previously[10]. The choice of additional instruments will vary depending on the study aims; however, at least mood states and the severity of somatic impairment should be evaluated.

The SSAS can be useful as an indicator of somatization in the therapy of a broad range of disorders, from impaired self-awareness [37-39] to various psychiatric disorders[40,41]. The concept of somatosensory amplification helps patients and physicians to better understand situations in which the psychiatric symptoms do not match the patients' clinical conditions and also provide useful information for choosing the appropriate pharmacological or psychological therapy. The SSAS would be useful in the treatment of patients with specific psychosomatic illness (e.g., irritable bowel syndrome[37,42,43] and chronic pain[22,44-46]), psychiatric disorders (e.g., somatoform disorders [47-52], anxiety disorders[53,54], and mood disorders[50,55]), stress reaction (e.g., reaction to bereavement[56] and other important psychosocial events[55]), and medical disorders (e.g., infectious disease[56] and heart disease[57,58]).

## Conclusion

A total of 50 English-language articles[8,10,11,13-15,20,22-29,33-55,57-68] were identified using the text words "somatosensory amplification" through a MEDLINE search from 1966 to April 2007. Somatization is a common feature in patients with mind/body distress, and the concept of somatosensory amplification provides a new approach to psychosomatic research [69-71]. It can help us to identify the explicit factors mediating the links between somatic and psychological symptoms. While somatosensory amplification has a role in the presentation of somatic symptoms, it is closely associated with other factors, namely, anxiety, depression, and alexithymia that may also influence the same. The specific role of somatosensory amplification with regard to both neurological and psychological function should be clarified in future studies.

## Abbreviations

BAEP: Bainstem auditory-evoked potential.

DSM-III: Diagnostic and statistical manual of mental disorders, third edition.

DSM-III-R: Diagnostic and statistical manual of mental disorders, third edition, revised.

DSM-IV: Diagnostic and statistical manual of mental disorders, fourth edition.

DSM-IV-TR: Diagnostic and statistical manual of mental disorders, fourth edition, text revision.

EEG: Electroencephalography.

ERP: Event-related potential.

SD: Standard deviation.

SSAS: Somatosensory amplification scale.

SSEP: Short-latency somatosensory evoked potential.

TAS: Tronto Alexithymia Scale

VEP: Visual evoked potential.

## Competing interests

The author(s) declare that they have no competing interests.

## Authors' contributions

MN wrote the first draft of the paper, and AJB revised it critically for important intellectual content. Both authors read and approved the final manuscript.
